# Serving by choice: Motivations and determinants of voluntary military reservism in Spain

**DOI:** 10.1371/journal.pone.0359544

**Published:** 2026-10-05

**Authors:** José Baqués, Vanesa Berlanga, José M. Pina, Franciele Corti

**Affiliations:** 1 Department of Political Science, Constitutional Law, and Philosophy of Law, Universitat de Barcelona, Barcelona, Catalonia, Spain; 2 Department of Education and Humanities, Universitat Abat Oliba CEU, CEU Universities, Barcelona, Catalonia, Spain; 3 Department of Marketing Management and Market Research, Universidad de Zaragoza, Zaragoza, Aragon, Spain; Federal University of the ABC: Universidade Federal do ABC, BRAZIL

## Abstract

Military reserves are an essential component of any country’s defence system. This study analyzes the Spanish model of voluntary reservists, involving over 3,000 citizens who temporarily join the Armed Forces to train and serve in areas related to their expertise. Using a territory-level dataset covering 2020–2024, the article integrates perspectives from rational choice theory, new institutionalism, public service motivation, and gender studies into a multi-layered explanatory framework. Results indicate that economic and institutional variables are systematically associated with regional differences in reservist numbers, while symbolic factors—proxied through online salience—show mixed but suggestive evidence. The analysis also identifies trends within the reservist population, including increasing female participation, aging, and a limited activation rate. Overall, the findings support a multidimensional model of engagement and offer insights for recruitment strategies, gender integration debates, and civil–military relations in democratic societies.

## Introduction

The military reserve constitutes a strategic and essential component of the armed forces, supporting active units in contexts of emergency, international crises, or extraordinary operational needs. In a scenario marked by hybrid threats and increasing global insecurity, countries like Spain have recognized the importance of maintaining a well-prepared and readily available military reserve [1]. However, this model remains comparatively less developed than those implemented in other NATO countries. In this paper, ‘strategic’ is used in a functional and doctrinal sense (what reserves are expected to provide), not as a claim that the Spanish VR system has been a sustained policy priority, which is reflected in its low activation rates, organizational constraints, and aging reservist profile, as shown later in this study.

Although the Spanish case may seem marginal in an international security framework, its limited political and social visibility makes its study particularly relevant. Analysing an institutionally weak model allows for the identification of structural and regulatory barriers that may also be present in other underdeveloped reservist systems. Furthermore, Spain represents a paradigmatic case of the civil–military gap, offering analytical insights that may be relevant to other European contexts facing similar challenges in integrating society into national defence.

Rather than treating Spain as a representative benchmark, we approach it as a low-visibility, institutionally constrained (‘hard’) case, useful for identifying the barriers and frictions that can limit the development of voluntary reserve systems in democratic settings.

Due to its strategic relevance, the Spanish reserve has been analysed by official bodies [2–4] and has become the subject of an emerging debate that highlights both its strengths and limitations, particularly regarding its operational capacity, retention ability, and adaptability to contemporary threats [1,5,6]. In Spain, a particularly debated model is that of the voluntary reservist.

For the purposes of this study, a “voluntary reservist” (hereinafter, VR) is defined as a Spanish citizen who, after successfully completing a selection process, signs a temporary commitment with the Spanish Armed Forces (SAF) to periodically integrate into operational units. This category differs from the compulsory reserve—currently deactivated—and from the special availability reservists, who are former professional military personnel placed in reserve status after completing their long-term commitment.

In the academic field, the VR model has received only limited attention [7], while reservism as such is widely recognized for its strategic and social value [8]. In particular, the Spanish case remains comparatively neglected. Existing studies are fragmented and often outdated [9–13], while more recent research highlights that voluntary reservists continue to lack institutional recognition and are undervalued by both civilian and military leaders, despite their potential to reinforce the society–defence bond [12]. This imbalance reveals a clear gap in the literature, calling for updated empirical analysis and a systematic theoretical framework to better understand the Spanish VR system.

This study seeks to address that gap by providing updated empirical evidence and an analytical framework combining rational choice theory, new institutionalism, and public service motivation, together with a gender perspective. Using official data for 2020–2024, it examines the extent to which economic conditions, institutional factors, and social-symbolic motivations are associated with the characteristics and territorial distribution of voluntary reservists across Spain. In doing so, the study advances the limited empirical literature on voluntary military reservism by moving beyond a purely descriptive characterization of the Spanish case.

Specifically, the present work is guided by the following question: What structural, institutional, and social factors are associated with the current characteristics and territorial distribution of the voluntary reservist (VR) system in Spain, and what implications may be derived for its future development? To answer this, the study draws on data collected by the Spanish Armed Forces between 2020 and 2024, complemented with secondary sources, and examines:

- The recent evolution of the model in demographic, administrative, and functional terms;
- The territorial implementation of the model, examining the distribution of reservists across autonomous communities in relation to both the civilian and military population;
- The association between economic factors (related to income and employment), institutional factors (military presence), and social-symbolic factors (public perception of the SAF and interest in the VR figure) on the number of voluntary reservists;
- The main challenges faced by the system, such as the aging of personnel or voluntary resignations;
- And the opportunities for improvement, based on a comparative analysis with models from allied countries such as the United States, France, and Poland, selected for their structural differences and their relevance within the NATO context.

This study starts from the premise that the Spanish VR system faces structural, regulatory, and organizational constraints that may affect its development and operational effectiveness. Building on this premise, the theoretical framework developed below leads to three hypotheses examining the economic, institutional, and social-symbolic factors associated with territorial variation in voluntary reservist participation.

Overall, the paper seeks not only to contribute to academic knowledge about the role of reservists in the defence system but also to generate proposals aimed at designing more effective, inclusive, and forward-looking public policies adapted to the demands of the 21st century. Ultimately, it evaluates how far the current Spanish model is aligned with the operational, technological, and civic demands posed by the evolving strategic environment.

## Theoretical framework

### The reserve in armed forces

We can define the reserve as “men and women who combine civilian working life with different forms of duty in the armed forces” [14, p. 285], and who are mobilized in times of crisis [7]. They have also been described as “transmigrants” who journey between the military and civilian spheres depending on need [15], a metaphor that has since been expanded to include the implicit contracts that reservists continuously negotiate with the military and the state [16]. Existing research points to the challenges career military personnel face when returning to civilian life after retirement, including loneliness and social isolation among both service members and their families [17–19], while also indicating that female veterans may confront additional complexities during reintegration into civilian life [20]. In this sense, reservists—both men and women—may be comparatively less vulnerable to these consequences given their continued integration into civilian society.

The concept of military reserve dates back to the 17th century, with its roots in European citizen militias and the defensive practices developed in the British colonies of North America [8,21]. During the 20th century, and especially in the context of the Cold War, reserves took on a strategic role as a means of maintaining an immediate response capacity in the face of potential military escalation [22].

This approach aligns with the contributions of Griffith, who emphasizes how reserves constitute a tool of strategic resilience in hybrid scenarios [8], and with the work of Ben-Ari & Connelly, who highlight the importance of adapting reserve models to new technological and social threats [7].

Following in the footsteps of the United Kingdom, which abolished compulsory military service in 1963, most Western countries have gradually professionalized their armed forces, opting for fewer personnel with higher efficiency to carry out missions both domestically and abroad. This paradigm shift led to greater interest in reservist models as a means of complementing existing capabilities [8].

The integration between professional forces and reserves is not without tensions. The U.S. Total Force project of 1969, which aimed for full integration between professional and reserve units, quickly revealed its flaws. The United States deployed some reserve brigades during the 1991 Gulf War, but the pre-deployment activation periods were insufficient to bring them up to the level of professional forces. Nevertheless, reservists played a key role in highly specialized support functions such as translation, medical assistance, logistics, and even military police duties [23].

Reservists are often referred to as *weekend warriors* in both the United States [24] and the UK [25]. In the Australian case, the term *koala* has even been used to refer to reservists: “a protected species not to be exported, not to be shot at” [26, p. 303]. However, some recent reports, applicable to the British case, note an improvement in this regard. Recent evidence, however, shows that perceptions of Reservists as less professional or committed persist within the UK forces, posing significant barriers to full integration [27].

In Israel, a country with a small population and a challenging security environment, attempts to integrate reservists into combat missions have also encountered difficulties, both on the front lines and in rear positions [28]. Aware of these challenges, countries such as the United Kingdom and Germany generally assign reservists to roles in logistics, CIMIC (civil-military cooperation), and internal security, often avoiding deployment in direct combat operations.

Despite their operational limitations, reservists provide added value to the armed forces. Due to their dual status as civilians and military personnel, they fulfil a legitimizing and visibility function that professional soldiers—focused on operations and with limited availability for public engagement—cannot always provide [23]. In a certain sense, the reservist becomes an ambassador of the Armed Forces, acting as a bridge between the military and society.

The theoretical framework of civil-military relations has been pointing to a phenomenon of increasing civilianization within the armed forces [29]. This process leads either to their fragmentation according to the branch of service and rank [30] or even to the emergence of a new mindset less oriented toward warfare [31]. Underlying all these perspectives is the penetration of postmodern values into military barracks, raising concerns about a widening gap between civilians and the military in each society. However, reserves can help reduce this gap.

Some experts in reservism have highlighted the impact of these social changes [28], but the issue deserves greater attention. The reserve makes it possible to establish two-way communication channels: for civilians, it contributes to the development of leadership, command, and discipline or resilience skills, and may also offer benefits in terms of social prestige, public recognition, and, under certain conditions, access to public service. For reservists, beyond their strictly military role, participation in the reserve enables them to act as “ambassadors of the Armed Forces to civil society” [14, p. 296], fostering greater visibility, social acceptance, and perceived legitimacy of the military within the broader societal context [7]. In this sense, the routine presence of reservists in civilian environments—such as workplaces, voluntary activities, and family settings—positions them as informal intermediaries between the armed forces and society at large [7].

Recent international literature also confirms the role of reservists as a key element in reducing the civil-military gap. The active presence of reservists in civilian environments has been shown to strengthen the social legitimacy of the Armed Forces and their connection with citizens [7,8].

### The Spanish reserve

In Spain, scholars concur that the voluntary reserve has remained politically invisible and has received limited institutional support [9–13]. Recent studies, however, also emphasize that both military and business leaders consider it essential to reinforce and expand this model in order to strengthen the society–defence link [12].

This limited institutional support contrasts with the prominence that reserves hold in other countries, some of which rely on compulsory models, also known as conscript reserves. These are relatively exceptional cases, such as Israel—“unique and without parallel among nations,” in the words of Ben Gurion [28, p. 218]—or Switzerland, described as “a small neutral state in the heart of Europe that historically faced a great security political change” [32, p. 242].

The extensive mobilization of the entire citizenry, even as reservists, is a national capability that few states possess. In the Spanish case, it is difficult to consider such a measure given the likely social rejection, as shown by scientific research. In 2022, barely 37.7% of Spaniards were willing to fight in a war (compared to 66.5% in 1984). In the U.S., that figure barely exceeded 60%, and in Germany, it was 52.5% [33]. Among the Western countries studied, only Sweden, with a strong reservist tradition, is slightly above 80%. In light of this situation, the Spanish Chief of the Defence Staff (JEMAD) has recently advocated for strengthening the voluntary reserve model, ruling out that, in the face of new threats, it is feasible to maintain purely professional armed forces or that it would be realistic to return to the universal compulsory military service model [34].

Historically, Spain relied on a “mass (mandatory) reserve” prior to the abolition of compulsory military service. By the late 1980s, the number of reservists who had completed the so-called *mili* (mandatory military service) reached 2.4 million people [12]. In Spain, Law 17/1999 introduced the professionalization of the armed forces, suspending compulsory service and redefining the role of the reserve as a flexible component adapted to modern demands. In parallel with France, the country abandoned conscription in 2001 in favour of a more efficient professional model for fulfilling missions both within and beyond national borders.

The legal framework governing the reserve system in Spain is based on the principles established by Organic Law 5/2005 on National Defence, which defines the missions of the Armed Forces (SAF) and, by extension, the role of the reserve. Law 8/2006 and Royal Decree 383/2011 detail the requirements, rights, and duties of reservists, establishing specific criteria for their selection, training, and activation. Within this framework, the Spanish system includes three categories: voluntary reservists, those with special availability, and mandatory reservists.

As mentioned in the introductory section, the group of voluntary reservists is made up of civilians who temporarily join the Armed Forces during activation periods, the duration of which is established each year in the Annual Activation Plans approved by the Ministry of Defence. In recent years, these periods have been allowed to last up to 5 months per year (4 months in service plus an additional month of training). However, Ministerial Order 56/2021 [35] specifies that time spent on service abroad is not deducted from this maximum limit, and Ministerial Order 47/2023 [36] authorizes the Undersecretariat of Defence to extend it in exceptional cases through an express resolution.

In contrast to voluntary reservists, reservists with special availability are enlisted personnel from the ranks of troops and seamen who have reached the age of 45 and have completed their long-term commitment. This group may also include complementary officers—usually captains or lieutenants—who complete their commitment after 18 years of service. Finally, mandatory reservists are Spanish citizens between the ages of 19 and 25 who, in the event of an exceptional situation, would be declared as such by the Government.

Organizationally, the Spanish reserve is designed to complement active forces, contributing value both in day-to-day needs and in extraordinary situations requiring a rapid response. The latter includes both domestic missions—such as disaster relief—and international ones, such as peacekeeping operations. However, in practice, the reserve faces several challenges, including a low activation rate—virtually non-existent in international missions—and training deficiencies [4].

Despite the VR model having been operating in Spain for more than 20 years, data from Center for Sociological Research (CIS) surveys conducted between 2009 and 2017 [37–41] and the June 2022 barometer [42] shows that between 5% and 10% of the population was unaware of this role, while nearly 70% considered it very unlikely that they would become a reservist in the future [41]. This lack of awareness and social appeal may be partly due to the limited number of activations over the years. As will be shown in the empirical section, most of these activations also tend to be concentrated in non-specialized areas [4], which represents an underutilization of the available potential and contributes to the demotivation of many reservists, who expect to perform more meaningful roles.

Closely linked to low mobilization is the absence of a coherent strategy for continuous training and professional development. Training is often limited to basic and sporadic sessions, hindering preparedness for complex challenges such as cybersecurity and intelligence operations. This issue partly results from a mismatch between current legislation and the operational needs of the strategic environment [3,4,43,44]. For example, the rise of hybrid threats and advances in cybersecurity demand legislative updates that would allow for more specialized and flexible training for reservists.

Beyond these challenges, another structural weakness of the Spanish model concerns the lack of a gender perspective, as existing legislation does not include specific measures to promote female participation in operational and strategic roles, thereby limiting the diversity and adaptability of the system [12].

### The Spanish reserve compared to other countries’ models

Within NATO, reserve forces constitute a fundamental component of defence planning, contributing to force generation, operational resilience, and the integration of specialized civilian expertise during both military operations and domestic emergencies. Although reserve systems differ across member states according to their historical, legal, and strategic contexts, NATO promotes the development of interoperable and well-prepared reserve forces capable of complementing active personnel. Spain forms part of this common framework, although its voluntary reserve system has evolved more gradually than those of several allied countries.

In contrast to the Spanish model—where the reserve plays a relatively marginal role in the SAF—many NATO countries have adopted comprehensive approaches that assign reservists a more prominent place in national defence [21,22]. These countries have integrated their reservists more effectively through sustained preparation, incentive mechanisms, and inclusive policies [7,8].

To provide an international benchmark, this study conducts a comparative analysis of the United States, France, and Poland. The selection of these three cases is deliberate: they are highly relevant within NATO, illustrate diverse approaches to reservism—full integration in the U.S., a mixed model with inclusive policies in France, and technological specialization in Poland—and offer lessons that may be applied to the Spanish context.

The contrast with Spain is especially evident in activation rates. Whereas fewer than 20% of Spanish reservists are activated each year [4], more than 60% of U.S. reservists participate annually in operational exercises or real missions [45]. In the United States, reservists play a central role in both national and international operations, from combat deployments to crisis response. This is supported by rigorous, recurring training—including monthly drills and annual exercises—that ensures immediate readiness [45]. Economic incentives, such as retention bonuses and educational benefits through the GI Bill, further contribute to both recruitment and retention [21]. By contrast, the Spanish model lacks comparable levels of activation and international engagement. Addressing this gap would require the establishment of systematic training cycles and the introduction of more competitive incentive schemes to improve preparedness and commitment.

France has implemented targeted policies to attract young people and women, achieving a female participation rate of 17%, higher than the European average; currently, 24% of candidates who pass the officer selection exams are women [46]. Recruitment campaigns aimed at students and young professionals, coupled with job flexibility and specialized training in technological and logistical fields, have broadened the appeal of the French reserve. This case illustrates how inclusive strategies can enhance both operational effectiveness and public legitimacy—an area where Spain could benefit by reducing gender disparities and increasing the attractiveness of the reserve among younger generations.

Poland, meanwhile, has prioritized resilience against hybrid threats by creating specialized cyber defence units tasked with protecting critical infrastructure and countering disinformation campaigns [22]. Polish reservists also receive advanced training in logistics, ensuring efficient mobilization in military operations. This reflects the broader regulatory emphasis in Poland and France on technological readiness and the incorporation of reservists into specialized units.

The comparative evidence shows that Spain can draw valuable insights from allied countries that have optimized their reserves through innovative strategies. Overall, these cases underscore the limitations of the Spanish model while also pointing to potential avenues for reform. Robust training structures in the U.S., France, and Poland contrast with Spain’s irregular and often insufficient training system. The well-established incentive mechanisms in those countries further highlight the absence of adequate stimuli to retain talent in Spain. Likewise, strong commitments to gender inclusion and youth engagement abroad emphasize persistent gaps in the Spanish reserve. Finally, the technological orientation of Poland’s system—focused on cyber defence and advanced logistics—marks an area where Spain should place greater emphasis to adapt to today’s strategic environment.

Ultimately, building on NATO standards, the modernization of Spain’s reserve system should adopt a comprehensive approach that strengthens not only operational capacity but also the public image of the reserve and its integration into national defence policy. Closer collaboration with international partners would also facilitate the exchange of best practices and the adoption of higher standards in reserve management [1].

### Motivations of reservists hypotheses

Various studies have shown that, as with other professions, professional military personnel are motivated by a range of external and internal factors. In a pioneering work, Janowitz described military personnel as “professional citizens” exposed to societal norms, values, and demands [29]. Later, Moskos introduced the distinction between institutional and occupational models, emphasizing that military personnel’s motivation depends both on tangible rewards and on the recognition of their professional role [47]. These theories are fully applicable to reservists, as demonstrated by Tresch in the Swiss case [32]. According to this author, many reservists adopt a cost-benefit logic, evaluating the effort required and the compatibility of military commitments with their professional and personal lives. In this regard, the citizen-soldier paradigm or the notion of duty may be losing strength as primary motivational factors, with utilitarian criteria increasingly dominating individual decisions [1,32].

Taking Spain as a case study, this article draws on rational choice theory [48,49], new institutionalism [50], and the concept of public service motivation [51], while also incorporating a gender perspective, to build a framework for understanding voluntary military reservism. It argues that the number of citizens joining the voluntary reserve in a specific region may reflect the combined influence of three dimensions: structural opportunity, institutional presence, and symbolic or motivational engagement.

From a structural perspective, rational choice models emphasize cost-benefit calculations in individual decision-making. Indeed, the demand for military manpower has long been analysed through economic determinants such as income levels, unemployment, and opportunity costs [52,53]. Citizens may be more likely to join the reserve in contexts of lower income or higher unemployment, where the relative value of military affiliation is greater [49]. Both job opportunities and relative wellbeing have traditionally influenced the decision to voluntarily enlist in the armed forces [48].

Following the cost-benefit logic described by Tresch, in regions where the economic situation is more precarious, there may be a greater incentive to pursue voluntary reservist status [32]. Hence, prior research has found a relationship between economic precarity and enlistment in reserve or auxiliary forces in several countries [53]. In some cases, the earnings can exceed those from civilian jobs; in others, reservists may combine this activity with their civilian employment. Moreover, some may regard it as a pathway to accessing other civilian or public sector positions.

Institutional factors, drawn from new institutionalist theory, relate to the accessibility and visibility of military infrastructure and support. Where military units, bases, or administrative offices are present, citizens may view military service as more accessible and legitimate [50,54]. Military personnel are not a social group detached from society but rather are fully integrated into it [29]. Therefore, the existence of military facilities in a given area can lead citizens to perceive military service as a legitimate and accessible option [1]. Another potential mechanism linking military presence and voluntary reservist enlistment is the concentration of military veterans and former service members in specific regions. Previous research has shown that military service often extends across generations, with family traditions influencing attitudes toward military careers and public service. Consequently, territories with a stronger military legacy may exhibit higher levels of reservist participation, not only because of the presence of active-duty personnel but also due to the influence of veteran communities and family networks. Additionally, the number of voluntary reservist (VR) positions offered depends on the needs of the various military units in each region. Although Spanish reservists may serve in units other than those originally assigned, the institutional military presence in a territory could be a major factor in attracting local citizens.

Finally, symbolic and motivational dimensions draw on the concept of the citizen-soldier [29] and theories of public service motivation [51]. Individuals may be drawn to voluntary service not just for instrumental reasons, but as an expression of civic duty, identity, and alignment with national values. According to Tresch, many reservists adopt a utilitarian logic, weighing military commitments against personal and professional life [32]. While traditional notions of military duty are losing prominence, new motivations rooted in civic participation and public service are becoming increasingly relevant. Similar to private companies, public institutions with a favourable image tend to foster more positive attitudes and behaviours among citizens. A positive perception of the SAF—and specifically of the VR figure—will directly influence recruitment.

This triad of factors (structural, institutional, symbolic) is examined in this study through different variables. The structural layer is captured through income per capita and unemployment rates. The institutional layer is operationalized via the presence of active-duty military personnel in each autonomous community. The social-symbolic layer includes both public perception of the Armed Forces and expressed interest in reservist service, the latter measured via Google Trends. By using this three-part approach, the study may offer a more nuanced understanding of what drives voluntary enlistment in the Spanish context. Although both dimensions relate to the social legitimacy of the Armed Forces, the institutional dimension refers to direct exposure to military organizations through the physical presence of units, personnel, and military infrastructure, whereas the symbolic dimension refers to citizens’ attitudes, values, perceptions, and expressed interest in military service. Distinguishing between these two dimensions allows us to assess whether enlistment is more closely associated with opportunity structures and organizational proximity or with broader patterns of social legitimacy and civic engagement. Conceptually, we treat enlistment outcomes as the result of a joint supply–demand process: institutional presence reflects the availability and salience of opportunities (supply/access), while economic and symbolic variables capture contextual incentives and engagement (demand/attitudes). Building on this theoretical framework, the following hypotheses are proposed:

H1 (Structural factors): Regions with (a) lower income levels and (b) higher unemployment rates will exhibit higher rates of voluntary reservist enlistment.
H2 (Institutional factors): Regions with greater presence of Spanish Armed Forces (SAF) personnel will exhibit higher rates of voluntary reservist enlistment.
H3 (Symbolic factors): Regions with (a) more favourable public perceptions of the SAF and (b) higher interest in reservist service will exhibit higher rates of voluntary reservist enlistment.

## Materials and methods

To systematically analyse the voluntary reservist (VR) model in Spain, a quantitative study was conducted, consisting of a first descriptive part and a second part estimating a panel data model. In both cases, the selected time frame is the period from 2020 to 2024, as it represents the most recent interval with consolidated and continuous data published by the Spanish Ministry of Defence. This period also allows for the observation of current trends following the organizational effects of the pandemic and within an unstable geopolitical context in Europe.

The descriptive analysis presents the evolution of the VR figure over the five years studied, focusing on key sociodemographic patterns, as well as functional and territorial distribution.

The data collected by the Spanish Ministry of Defence include distribution by military branches (Army, Air and Space Force, Navy, and Common Corps), demographic variables (average age, age groups, gender distribution), administrative status (activated and available), unemployment and assigned specialty, as well as geographic information (provinces of origin and assignment).

In line with the theoretical framework, the second part of the analysis examines the territorial distribution of reservists in each Spanish autonomous community using three types of explanatory variables: structural factors, institutional presence, and social-symbolic engagement.

Structural factors were operationalized through economic indicators. It is hypothesized that the size of the reserve in each territory may be associated with the economic conditions of that region, as citizens in more disadvantaged areas may be more attracted to the opportunity of becoming a VR as a potential source of income. For operationalization, data from the National Statistics Institute (INE) from 2019 to 2023 were used, calculating two average variables: one for per capita income and another for employment. From the same source, population data for each autonomous community (year 2021) was collected to be used as a control variable.

Institutional presence was measured through military personnel distribution. It is expected that the institutional presence of the Armed Forces (SAF) in each region could influence the availability and legitimacy of reservist opportunities. The total number of career military personnel assigned to each Spanish region was considered an indicator of the military’s presence. This data was obtained from statistics published by the Ministry of Defence between 2020 and 2024, calculating the average value over that period.

Social-symbolic engagement was proxied through public perception and online interest. Data on the public image of the SAF was used, obtained in 2019 from the study “Sociological Observatory on Public Perception of the Activities, Plans, and Programs of the Culture and Awareness of Defence Plan” [55]. This study surveyed 3,000 citizens aged 16 and over, with a sampling error of less than 2%. Additionally, internet search frequency for the term “voluntary reservists” (in singular and plural) was considered as a proxy for online salience/attention to voluntary reservism. This proxy may reflect interest but can also capture curiosity or media-driven attention; it is therefore interpreted cautiously. These data were extracted from Google Trends, a tool considered suitable for assessing attitudes toward an entity based on Google search activity [56,57].

Regarding the main dependent variable of the study—the number of voluntary reservists—the data were obtained from the publicly available annual statistical reports published by the Spanish Ministry of Defence for the period 2020–2024. These official statistics are compiled by the Ministry from administrative data recorded in the SIPERDEF personnel information system, managed by the Directorate General of Personnel. Across the five annual reports, the reported number of voluntary reservists totals 15,225, corresponding to approximately 3,000 voluntary reservists per year [3,4,43,44,58]. The authors did not have direct access to SIPERDEF or to any confidential or personally identifiable individual-level data. Special availability reservists were excluded from the analysis, as they constitute a different category.

## Results

### Profile of voluntary reservists (VRs)

According to data collected by the Ministry of Defence, between 2020 and 2024 there was a 4.6% decrease in the number of voluntary reservists (VRs), resulting in a total of 2,969 personnel by the end of 2024 (Table 1). Compared to VRs, it is worth noting that the number of special availability reservists (former professional military personnel) almost tripled, from 3,173 to 9,520 during the examined period.

**Table 1:** Profile Summary of Reservists, 2020–2024.

Year	Total VRs	Available status*	Activated	Mean activation time per activated reservist (days)	Women	Average Age
2020	3,112	49.5%	3.1%	N.D.	16.3%	50.1
2021	3,123	40.6%	46.1%	62.1	16.8%	50.3
2022	3,031	33.5%	51.9%	61.8	17.7%	50.0
2023	2,990	27.9%	55.4%	66.7	18.4%	50.2
2024	2,969	23.8%	54.9%	70.0	19.8%	50.3

Source: Own elaboration based on data from the Spanish Ministry of Defence (2020–2024).

* Number of VRs relative to the total number of reservists, including those with special availability status.

Excluding the year 2020, marked by the pandemic, the number of VRs activated annually has been roughly half of the total. On average, each reservist is activated for about two months per year, with the average duration of each activation being approximately 40 days.

In sociodemographic terms, the majority of reservists are male, although the proportion of female participants has been gradually increasing, rising from 16.3% in 2020 to 19.8% in 2024. The group is characterized by an aging profile: while the average age of special availability reservists is around 47, the average age among voluntary reservists reaches 51.

In line with other countries, the Spanish Armed Forces (SAF) are structured according to their major domains of operation: the Army, the Air and Space Force, and the Navy. Additionally, the Common Corps serve all three branches in areas such as legal affairs, intervention, healthcare, and military bands. As shown in Table 2, all branches include reservists, although nearly half are assigned to the Army (46.9% in 2024).

**Table 2:** Distribution of Voluntary Reservists (VRs) in the Spanish Armed Forces, 2020–2024.

Year	Army	Air and Space Force	Navy	Common Corps
2020	51.3%	14.0%	19.9%	14.8%
2021	49.3%	14.2%	19.5%	16.9%
2022	47.4%	14.6%	18.6%	19.3%
2023	47.6%	14.0%	17.3%	21.1%
2024	46.9%	14.0%	16.4%	22.7%

Source: Own elaboration based on data from the Spanish Ministry of Defence.

Going into further detail, Table 3 shows the rank (officers, non-commissioned officers, enlisted personnel) and specialties of Spanish voluntary reservists (VRs). For simplicity, the analysis focuses on the year 2024, and some categories have been grouped (for example, Engineering includes “Cultural Engineering,” “Logistics Engineering,” and “General Staff Engineering”).

**Table 3:** Rank and Specialty of Voluntary Reservists (VRs), 2024.

Assignment Area	Total	Officers	NCOs	Troops & Sailors
General Area (GHs)	1,048	58.7%	32.9%	8.4%
Logistics	708	22.3%	47.7%	30.0%
Medical	588	100.0%	0.0%	0.0%
Operations	248	50.0%	29.0%	21.0%
Cultural Affairs	135	87.4%	11.9%	0.7%
Engineering	115	97.4%	2.6%	0.0%
Legal Area	49	100.0%	0.0%	0.0%
Logistics Management	41	97.6%	2.4%	0.0%
Military Music	37	2.7%	97.3%	0.0%

Source: Own elaboration based on data from the Spanish Ministry of Defence. GHs: General Headquarters.

According to the data, one in three reservists (1,048) was assigned in 2024 to various tasks at general headquarters, without being classified under a specific specialty, and the majority of them (58.7%) were officers. Regarding specific areas, the most prominent was logistics (excluding engineers), with 708 reservists, mostly non-commissioned officers (47.7%) and enlisted personnel (30%). Next in line were healthcare specialists from the Common Corps, followed by specialists in Operations, Culture (general area), and Engineering—areas primarily composed of officers.

It is important to note that these data do not precisely reflect the actual professional profile of the VRs, but rather the specialty to which they have been administratively assigned. For example, healthcare personnel are not exclusively found in the Common Corps; they are also distributed across the three branches of the Armed Forces, serving in various units.

### Territorial distribution of voluntary reservists (VRs)

Spain is territorially organized into 17 autonomous communities and 2 autonomous cities (Ceuta and Melilla). Some official designations may cause confusion for international readers, as terms such as “Basque Country” or “Valencian Community” do not imply a status equivalent to that of an independent state, but rather reflect the internal administrative nomenclature.

As shown in Table 4, 27% of all voluntary reservists come from Andalusia, which contributes the highest number, followed by Madrid, Castile and Leon, and the Valencian Community. The number of VRs appears to correlate with the number of career military personnel and the population size of each region, although some exceptions are evident. For instance, in the case of Madrid, the number of reservists is 10 percentage points lower than that of career military personnel, yet it aligns with the region’s overall population size. The opposite is observed in Catalonia, where the proportion of career military personnel is significantly lower than that of reservists (1.1% vs. 6.2%), while reservists account for only a modest fraction of the overall population.

**Table 4:** Territorial Distribution of Voluntary Reservists (VRs).

Territories	VR (2024)	SAF Presence (2024)	Population (2021)	Income (€) (2019–23)	Unemployment (2019–23)	SAF Image (2019)	VR Interest (2019–23)
Andalusia	27.2%	20.5%	17.9%	26,539.6	20.5	7.1	43
Madrid	17.9%	28.2%	14.2%	38,621.4	11.5	7.0	41
Castile & Leon	8.1%	6.8%	5.0%	29,553.8	11.6	6.6	40
Valencian Community	7.5%	5.2%	10.7%	28,415.4	14.3	6.7	23
Catalonia	6.2%	1.1%	16.4%	35,676.8	11.2	4.6	13
Canary Islands	5.2%	6.2%	4.6%	27,135.2	20.5	7.0	34
Galicia	4.9%	5.8%	5.7%	28,803.6	12.1	6.4	26
Castile-La Mancha	5.0%	2.2%	4.3%	27,092.8	16.1	6.9	41
Murcia	4.2%	5.6%	3.2%	27,530	15.0	7.5	44
Aragon	3.1%	7.8%	2.8%	32,295	10.4	7.2	35
Balearic Islands	2.5%	0.9%	2.5%	32,794	18.1	6.2	23
Asturias	2.0%	0.7%	2.1%	29,689.2	13.7	6.9	26
Ceuta & Melilla*	1.7%	4.4%	0.4%	35,687.6	24.7	8.3	93
Extremadura	1.6%	3.1%	2.2%	23,637	21.4	7.1	26
Cantabria	1.2%	0.1%	1.2%	31,171	11	7.1	23
Basque Country	1.1%	0.9%	4.7%	38,471.2	9.3	4.2	10
Navarre	0.5%	0.4%	1.4%	38,254.8	10.2	5.4	17
La Rioja	0.1%	0.3%	0.7%	31,595	11.1	6.9	28

Source: Own elaboration based on data from the Spanish Ministry of Defence, the National Statistics Institute (INE), and Google (Google Trends).

Data for Ceuta and Melilla are aggregated. “SAF Presence” refers to career military personnel; “SAF Image” ranges from 0 to 10, and “VR Interest” (Google searches related to VR) from 0 to 100.

Economic and symbolic factors also appear to play a role. Ceuta and Melilla stand out for having both the highest public approval of the Armed Forces (an average score of 8.3 out of 10) and the greatest volume of online searches for “voluntary reservists” (indexed at 93 on a 0–100 Google Trends scale). Despite facing the country’s highest unemployment rates (24.7%), they rank among the most prosperous regions in terms of per capita income. This paradox supports the idea that structural opportunity is not solely driven by income, but by the relative value of reservist status in each context. In contrast, regions like the Basque Country and Catalonia score low on both public perception and online interest, suggesting weaker symbolic engagement.

To assess how these structural, institutional, and symbolic factors statistically influence the number of VRs, a panel data model was estimated. The dependent variable is the number of reservists in each autonomous community (i) over five years (t), from 2020 to 2024. Explanatory variables include military presence (active-duty personnel), public perception of the Armed Forces, interest in the VR figure (Google search trends), per capita income, unemployment, and population.

Due to the limitations of the sample size (90 observations, corresponding to 18 territories over 5 years) and the lack of time-varying data for some variables, the independent variables were defined as time-invariant but varying by territory. Accordingly, the model is best understood as a territorial association model using repeated annual observations, primarily aimed at accounting for cross-regional differences rather than within-region temporal dynamics. Thus, the model was specified as follows: ${VR}_{it}=\alpha_{it}+\beta_{\text{1}}{SAFPresence}_{i}+\;{\beta_{\text{2}}{SAFImage}_{i}+\beta_{\text{3}}{VRInterest}_{i}+\beta_{\text{4}}{Income}_{i}+\beta_{\text{5}}{Unemployment}_{i}+\beta_{\text{6}}{Population}_{i}+\varepsilon }_{it}\;$.

We acknowledge that some relationships—particularly between SAF presence and VR numbers—may involve reverse causality or common institutional drivers, and therefore the estimates are interpreted as associations, not causal effects. The model was estimated using the software Gretl (GNU Regression, Econometrics and Time-series Library), version 2024b. Specifically, the groupwise weighted least squares (WLS) method was applied. This method is recommended to correct for heteroscedasticity issues, which were detected using the Chi-square statistic (*p* < 0.001) [59]. Additionally, due to the variance in measurement scales, the models were estimated using standardized versions of the variables instead of their original raw forms.

Model estimates (Table 5) confirm that institutional presence (β = 0.34; p < 0.0001) and population size (β = 0.50; p < 0.0001) are the most influential factors, suggesting that access and logistical proximity play a major role in recruitment. These results provide strong support for H2.

**Table 5:** Panel Data Model Estimation.

	Coefficient	Standard Error	z	p
Constant	−0.11	0.01	−16.24	<0.0001***
SAF Presence	0.34	0.02	17.45	<0.0001***
SAF Image	0.01	0.01	1.12	0.26
VR Interest	0.05	0.01	4.73	<0.0001***
Income	−0.09	0.01	−8.55	<0.0001***
Unemployment	0.02	0.00	4.27	<0.0001***
Population	0.50	0.02	25.07	<0.0001***

Source: Own elaboration based on results obtained using Gretl. ***p < 0.001.

Structural variables also yield significant effects: lower income levels are associated with more reservists (β = −0.09; p < 0.0001), while higher unemployment is positively correlated (β = 0.02; p < 0.0001). This pattern supports both H1a and H1b, consistent with the rational-choice logic of the framework.

As for symbolic engagement, Google search trends are positively associated with VR numbers (β = 0.05; p < 0.0001), indicating that online behaviour may serve as a useful proxy for issue salience related to reservism. However, general public perception of the SAF shows no direct statistical effect (β = 0.01; p = 0.26), though its high correlation with search trends (r = 0.749; p < 0.001) suggests an indirect influence. Given the high correlation between SAF image and VR-related search salience, results for these variables are interpreted with caution as they may partly reflect overlapping informational environments. Therefore, H3a is not supported, while H3b is supported.

Together, these findings provide empirical support for a multidimensional model of reservist engagement in Spain. Structural, institutional, and symbolic factors all contribute—albeit unevenly—to shaping regional recruitment outcomes.

These findings are also consistent with the broader patterns observed across NATO reserve systems discussed earlier. The positive association between institutional military presence and reservist recruitment is in line with allied models that emphasize the integration of reserve forces into regular military structures. Likewise, the importance of economic and organizational conditions supports the view that effective reserve systems depend not only on individual motivation but also on sustained institutional investment, operational readiness, and long-term policy commitment. While Spain operates within the same NATO framework, the empirical evidence presented here suggests that its reserve system remains comparatively less developed in these dimensions.

## Discussion

The comparative cases are used as illustrative institutional benchmarks rather than directly comparable counterfactuals, given differences in scale, legal frameworks, and geopolitical exposure. Earlier research anticipated that reservists would play an important social role in reinforcing the legitimacy and visibility of the Armed Forces [23]. In the Spanish case, around 3,000 individuals from a wide range of professions voluntarily dedicate their time to supporting the needs of the military. However, they remain an underutilized resource, with relatively low activation rates and a concerning trend toward aging.

These conclusions are grounded in the empirical analyses of territorial distribution and activations conducted in this study, while the comparison with the U.S., France, and Poland provides an international context for interpreting the Spanish case [7,22,60]. Accordingly, the policy implications discussed below are limited to those directly supported by the empirical findings, particularly those related to territorial recruitment patterns, institutional military presence, socioeconomic conditions, and specific public interest in voluntary reservism.

Although the number of reservists declined between 2020 and 2024, more recent calls for applications suggest an increase. While the Armed Forces seek professionals from all fields, healthcare profiles are among the most in demand and are also the most likely to be activated in special circumstances such as natural disasters or overseas missions. However, the majority of reservists perform duties in less specialized areas such as administration or general logistics.

Voluntary reservists are distributed throughout Spain and, in proportional terms, are more widely represented than career military personnel. Regions with greater representation, such as Andalusia and Madrid, likely act as key hubs for the reserve system, facilitating both operational deployment and training. Nonetheless, the uneven territorial distribution identified in the analysis suggests that underrepresented regions may warrant particular attention when considering future recruitment and outreach strategies.

However, the present design focuses on explaining differences between territories rather than temporal changes in overall recruitment levels. Consequently, the results should not be interpreted as evidence regarding the causes of the nationwide decline in reservist numbers observed during the study period. The explanatory framework proposed in this study—which integrates structural, institutional, and symbolic dimensions—proves useful in interpreting these regional disparities. The panel data model shows that economic and institutional factors are significantly associated with the distribution of voluntary reservists. Specifically, citizens in economically disadvantaged areas appear more likely to enlist, validating the cost-benefit logic of rational choice theory [48,49]. Consistent with economics models of manpower demand, voluntary reservists in Spain may be understood not only as a civic phenomenon but also as part of a labour allocation mechanism within the defence sector [53].

Likewise, the presence of active-duty personnel is positively associated with greater visibility and perceived accessibility of military service [50,54]. On the symbolic front, while public opinion toward the Armed Forces does not have a direct statistical effect, the level of specific interest in the figure of the reservist—as measured by Google Trends—does. This nuance supports the idea that concrete, targeted awareness of reservism, rather than diffuse institutional reputation, plays a more decisive role in shaping individual motivation and engagement [7]. Similar findings emerge from international research, which shows that reservists’ prosocial motivation, understood as the willingness to serve others and society, significantly enhances the perceived meaning of military service and sustains their engagement [61].

### General implications

Overall, the analysis of the Spanish reservist model underscores the need to adapt this system to current strategic and operational demands. Hybrid threats, which combine conventional warfare with cyberattacks, disinformation, and psychological operations, have redefined the concept of security. Earlier research emphasized that the evolving nature of modern conflict requires a flexible and rapidly deployable military force [47].

In this context, reservists can play a key role in mitigating risks and, working alongside professional soldiers, help strengthen national resilience in the face of both internal and external crises. Increasing the number of activations appears to be a critical step toward enhancing response capabilities and ensuring that the reserve is a reliable and effective resource, even in support of frontline units.

With regard to policy implications, the empirical findings suggest that future strategies should focus particularly on the factors associated with territorial variation in voluntary reservist participation. The positive association between active-duty military presence and reservist numbers highlights the potential relevance of institutional visibility and proximity, while the significant association between specific interest in voluntary reservism and reservist presence suggests that targeted communication and awareness strategies may be more relevant than general perceptions of the Armed Forces.

The observed associations with income and unemployment further indicate that socioeconomic context should be considered when designing recruitment and retention strategies. Rather than applying uniform measures across the country, the territorial heterogeneity identified in this study suggests that policies may need to be adapted to regional conditions and recruitment environments.

Other issues identified descriptively in the study, including the aging of the reservist population and the gradual increase in female participation, remain relevant for the future development of the system. However, because the empirical model was not designed to identify their determinants or evaluate specific interventions, recommendations concerning training, gender integration, incentives, or broader organizational reforms should be addressed through further research.

Overall, the findings support a more evidence-based approach to the development of the Spanish voluntary reserve system, in which recruitment, communication, and territorial implementation strategies are informed by the institutional, socioeconomic, and social factors associated with reservist participation identified in this study. Beyond the number of reservists formally available, future assessments should also consider the capabilities they can provide, their level of readiness and availability, and their potential contribution to the operational requirements of the Armed Forces.

## Conclusion

This study has several limitations. First, some explanatory variables are time-invariant or averaged, which limits within-region inference and favours a cross-regional interpretation. Second, the analysis cannot fully disentangle institutional supply (quotas/allocations) from individual demand (applications), and potential endogeneity cannot be ruled out. A further limitation concerns the absence of regional data on military veterans and former service members. Since military traditions frequently extend across generations, veteran concentration may influence voluntary reservist recruitment through family and social networks. Because no homogeneous territorial data are currently available for Spain, this factor could not be incorporated into the empirical model and should be explored in future research. The research design employed allows us to account for territorial differences across autonomous communities but does not permit identification of the causes underlying the overall decline in reservist numbers observed in Spain during the study period. Examining this temporal trend would require additional longitudinal and individual-level data capable of capturing recruitment, retention, and withdrawal dynamics over time. Third, Google Trends captures online salience rather than verified enlistment intent. Consequently, the findings should be interpreted as evidence of statistically significant associations rather than causal relationships. While the observed patterns are consistent with the theoretical framework proposed, the study design does not allow causal inference regarding the factors associated with voluntary reservist enlistment.

In response to the research question, this study has confirmed the value of applying a multi-dimensional theoretical framework—integrating structural, institutional, and symbolic variables— to better understand the dynamics of voluntary military reservism in Spain. The analysis of the evolution of the voluntary reservist (VR) system between 2020 and 2024 reveals three key findings. First, the system is structurally normative but operationally weak, characterized by low activation rates, training deficiencies, and a progressive aging of personnel. Second, although female integration is growing, it remains limited in comparison to that of their male counterparts—a challenge that extends beyond the reserve to the Armed Forces (SAF) as a whole. Third, the territorial distribution of reservists follows an uneven pattern that does not always align with operational efficiency criteria. Overall, regulatory, organizational, and motivational barriers continue to limit the system’s actual effectiveness.

By operationalizing this tripartite model in empirical terms, the study also contributes to military sociology, civil-military relations, and public service motivation research. The integration of economic, institutional, and attitudinal indicators offers a more nuanced interpretation of regional disparities in reservist enlistment. The data obtained further highlights that broader public image may matter less than specific engagement with the reservist figure—an insight with implications for recruitment and communication strategies.

At the same time, several future lines of research could deepen our understanding of reservism. These include the study of reservists’ internal perceptions through surveys and qualitative interviews; analysis of the impact of recent legislative reforms on activation and retention levels; examination of the territorial efficiency of the reserve from a cost-effectiveness perspective; and a longitudinal evaluation of the effects of economic and gender incentives on personnel retention. A further line of research should examine the effective mobilization capacity of the Spanish reserve, moving beyond nominal personnel figures to assess readiness, availability, professional capabilities, integration with regular forces, and the number and type of reservists required under different operational scenarios.

Importantly, the findings reported here should not be interpreted as an explanation for the overall decline in voluntary reservist numbers observed in Spain between 2020 and 2024. Rather, they identify a set of structural, institutional, and symbolic factors associated with territorial variation in reservist distribution during that period. Ultimately, reservists represent not only a strategic asset but also a civic opportunity. Given the global relevance of adaptable and socially embedded reserve systems, the Spanish case may offer valuable insights for other countries seeking to modernize their military reserves in line with democratic values and evolving security demands. The reserve should no longer be regarded as a peripheral support structure, but as an integrated, skilled, and adaptable pillar of national defence—one that reinforces the civic dimension of defence in modern societies.

In short, studying the Spanish case makes it possible to understand the dynamics of reservist systems with low institutional visibility, making it a useful laboratory for proposing reforms that can be replicated in other countries in its environment. From an economic perspective, these results indicate that the Spanish voluntary reserve system can be seen as a cost-effective complement to professional forces, particularly in regions facing economic disadvantage. Future research should therefore assess not only the sociological and institutional dimensions of reservism, but also its efficiency and opportunity costs within the broader framework of defence economics.
